# Efficacy and safety of Jia Wei Bushen Yiqi formulas as an adjunct therapy to systemic glucocorticoids on acute exacerbation of COPD: study protocol for a randomized, double-blinded, multi-center, placebo-controlled clinical trial

**DOI:** 10.1186/s13063-020-04669-5

**Published:** 2020-09-03

**Authors:** Qing Kong, Shuming Mo, Wenqian Wang, Zihui Tang, Ying Wei, Yijie Du, Baojun Liu, Lingwen Kong, Yubao Lv, Jingcheng Dong

**Affiliations:** 1 Department of Integrative Medicine, Huashan Hospital, Fudan University, Shanghai, China; 2 Department of Integrative Medicine, North Hospital of Huashan Hospital, Fudan University, Shanghai, China

**Keywords:** Randomized clinical trial, Design and method, Acute exacerbation of COPD, Traditional Chinese medicine, Systemic glucocorticoids duration

## Abstract

**Background:**

Systemic glucocorticoids are effective for the management of chronic obstructive pulmonary disease (COPD) exacerbation but have serious adverse effects. Traditional Chinese medicine (TCM) can bring additional benefits to these patients but has few adverse effects. The present study aims to evaluate the efficacy and safety of Jia Wei Bushen Yiqi (JWBY) formulas in patients who suffer from COPD exacerbations and to investigate whether the short-term (5-days) systemic glucocorticoid therapy is non-inferior to the long-term (9-day) regime.

**Methods:**

In this multi-center, randomized, double-blinded trial, eligible inpatients with COPD exacerbation are randomly assigned to four groups (A, B, C, and D). Group A will receive placebo plus 5-day prednisone, group B will receive placebo plus 9-day prednisone, group C will receive JWBY formulas plus 5-day prednisone, and group D will receive JWBY formulas plus 9-day prednisone. The primary outcomes are the time interval to the patient’s next exacerbation during a 180-day following up and the COPD assessment test (CAT) during treatment. Secondary outcomes include lung function, TCM syndrome assessment, laboratory tests, and safety. The changes of the hypothalamic pituitary adrenaline axis (HPA axis) and inflammatory cytokine will be measured as well.

**Discussion:**

By demonstrating the advantages of utilizing TCM and an appropriate duration of systemic glucocorticoids, this effectiveness comparison trial will provide new references to physicians on how to improve the management of COPD exacerbation. The results of HPA axis and inflammation cytokine measurements will shed light on the molecular mechanisms and entail further mechanism studies.

**Trial registration:**

www.chictr.org.cn ChiCTR1900023364. Registered on 24 May 2019.

## Administrative information

Note: the numbers in curly brackets in this protocol refer to SPIRIT checklist item numbers. The order of the items has been modified to group similar items (see http://www.equator-network.org/reporting-guidelines/spirit-2013-statement-defining-standard-protocol-items-for-clinical-trials/).

**Table:** 

Title {1}	Efficacy and safety of Jia Wei Bushen Yiqi formulas as an adjunct therapy to systemic glucocorticoids on acute exacerbation of COPD: Study protocol for a randomized, double-blinded, multi-centre, placebo-controlled clinical trial TCMWSG: Traditional Chinese Medicine with Systemic Glucocorticoids
Trial registration {2a and 2b}.	ID: ChiCTR1900023364, registered on 24 May 2019. www.chictr.org.cn
Protocol version {3}	KY 2019-299,03,03, July, 2019
Funding {4}	This project was supported for 3 years to accelerate the development of Chinese medicine in Shanghai, China. [Grant No: ZY (2018-2020)-FWTX-4016], the National Natural Science Foundation of China (Grant No. 81704154), Chinese medicine innovation project of Shanghai Health Committee (No.ZYKC201601023) and National Natural Science Foundation of China (No.81501180)
Author details {5a}	Qing Kong^1#^, Shuming Mo^2#^, Wenqian Wang^1^, Zihui Tang^1^, Ying Wei^1^, Yijie Du^1^, Baojun Liu^1^, Lingwen Kong^1^, Yubao Lv^1^* Jingcheng Dong^1^* ^1^Department of Integrative Medicine, Huashan Hospital, Fudan University, Shanghai, China, ^2^Department of Integrative Medicine, North Hospital of Huashan Hospital, Fudan University, Shanghai, China *Correspondence: Yubao Lv, lvyubao80313@163.com *Correspondence: Jingcheng Dong, jingcheng_dong@yeah.net ^#^These authors have contributed equally to this project.
Name and contact information for the trial sponsor {5b}	Jingcheng Dong, jingcheng_dong@yeah.net
Role of sponsor {5c}	Qing Kong and Shuming Mo drafted the manuscript. Wenqian Wang, Zihui Tang, Baojun Liu, Yijie Du and Lingwen Kong participated in the design of the study. Ying Wei, Yubao Lv and Jingcheng Dong conceived the study, participated in its design and coordination, and drafted the manuscript. All authors read and approved the final manuscript.

## Introduction

### Background and rationale {6a}

Chronic obstructive pulmonary disease (COPD) will become the third leading cause of death worldwide in 2030 [1]. 13.7% of the population over 40 years old suffer from COPD in China [2], creating a large socio-economic burden [3–5]. COPD exacerbation is defined as the acute worsening of respiratory symptoms that require additional therapy [6–8]. Acute exacerbations of COPD impair pulmonary function and exponentially increase the risk of death [9]. Therefore, effective management of COPD is critical to human health.

According to international guidelines and evidence-based reviews, systemic glucocorticoids are recommended to treat COPD exacerbation [10–15]. The advantages include shortened recovery time and hospitalization duration, improved lung function and oxygenation, and reduced relapse risk and treatment failure, which have been demonstrated by numerous randomized clinical trials (RCT) [16–22]. However, the side effects like hypertension, hyperglycemia, gastrointestinal bleeding, psychiatric disease, and hypothalamic pituitary adrenal axis (HPA axis) suppression increase with the extension of treatment duration and the escalation of dose [23]. Controversy over the optional duration continues. On one hand, a dose of 40 mg prednisone (a common oral systemic glucocorticoid) daily for 5 days has been recommended by the Global Initiative for Chronic Obstructive Lung Disease (GOLD) Science Committee Report based on the REDUCE randomized clinical trial since 2015 [24]. The trial indicated the efficacy of 5-day systemic glucocorticoids is non-inferior to 14-day systemic glucocorticoids regarding relapse within a 6-month follow-up, but significantly reduced glucocorticoid exposure. On the other hand, a dose of 30–40 mg prednisone daily for 9–14 days [10, 12, 13] was suggested by another academy of China, Korea, and Europe in 2017. Yet, no clinical trials have determined the difference between the 5-day and 9-day regimes.

In addition, treatment individualization brings benefits. For instance, an inhaled corticosteroid (ICS) is more efficacious in patients with high blood eosinophils [25–27]. However, present pharmacotherapy has failed to reverse the downtrend in pulmonary function completely [28]. Hopefully, traditional Chinese medicines (TCM) can expand COPD treatment in terms of syndromic difference, also called Zheng [29]. Not only has TCM alleviated symptoms such as coughing, shortness of breath, and sputum for thousands of years, but also has demonstrated its efficacy and safety [30–34]. However, there are rarely studies focused on COPD patients during the acute exacerbation period, most of them focused on the relatively stable period.

We conducted a randomized and placebo-controlled trial enrolling stable COPD patients in 2014, which illustrated that TCM formulas called Bushen Yiqi (BY) formulas can improve the lung function, reduce the frequency of acute exacerbation of COPD, and modulate the HPA axis [35]. Dr. Shen replaced glucocorticoid therapy with TCM formula (BY) totally in chronic inflammatory disease [36]. Moreover, several ingredients in BY can decrease the inflammatory reactions in COPD animal models [37]. Recently, we have observed that BY formulae combined with another two Chinese herbs—Huang Qin (Scutellaria) and Chi Shao (Paeoniae Rubra Radix)—demonstrate more effectiveness on the management of acute exacerbation of COPD in clinical practice, such as relieving the symptoms including the cough, sputum, as well as shortness of breath. Interestingly, the laboratory experiments showed that the main compound of these two Chinese herbs benefits the animal of COPD model. For instance, *Scutellaria baicalensis* in Huang Qin significantly improved lung function, ameliorated the pathological damage, and attenuated inflammatory cytokines infiltration into the lungs [38]. Similarly, paeonol in Chi Shao showed anti-inflammatory and antioxidant effects against CS-induced lung inflammation in both in vivo and in vitro experiments [39]. Therefore, we propose that Jia Wei Bushen Yiqi formulae (JWBY)—Bushen Yiqi formulae combined with Huang Qin and Chi Shao—will benefit patients with acute exacerbation of COPD.

## Method {7}

This study aims to demonstrate non-inferiority of a 5-day therapy compared with a 9-day regimen of systemic glucocorticoids based on the COPD outcome during the 180-day follow-up period. It also seeks to determine the relative inferiority of JWBY formula as an adjunct treatment to systemic glucocorticoids compared with systemic glucocorticoids alone for COPD exacerbation.

### Trial design {8}

**Fig. 1:**
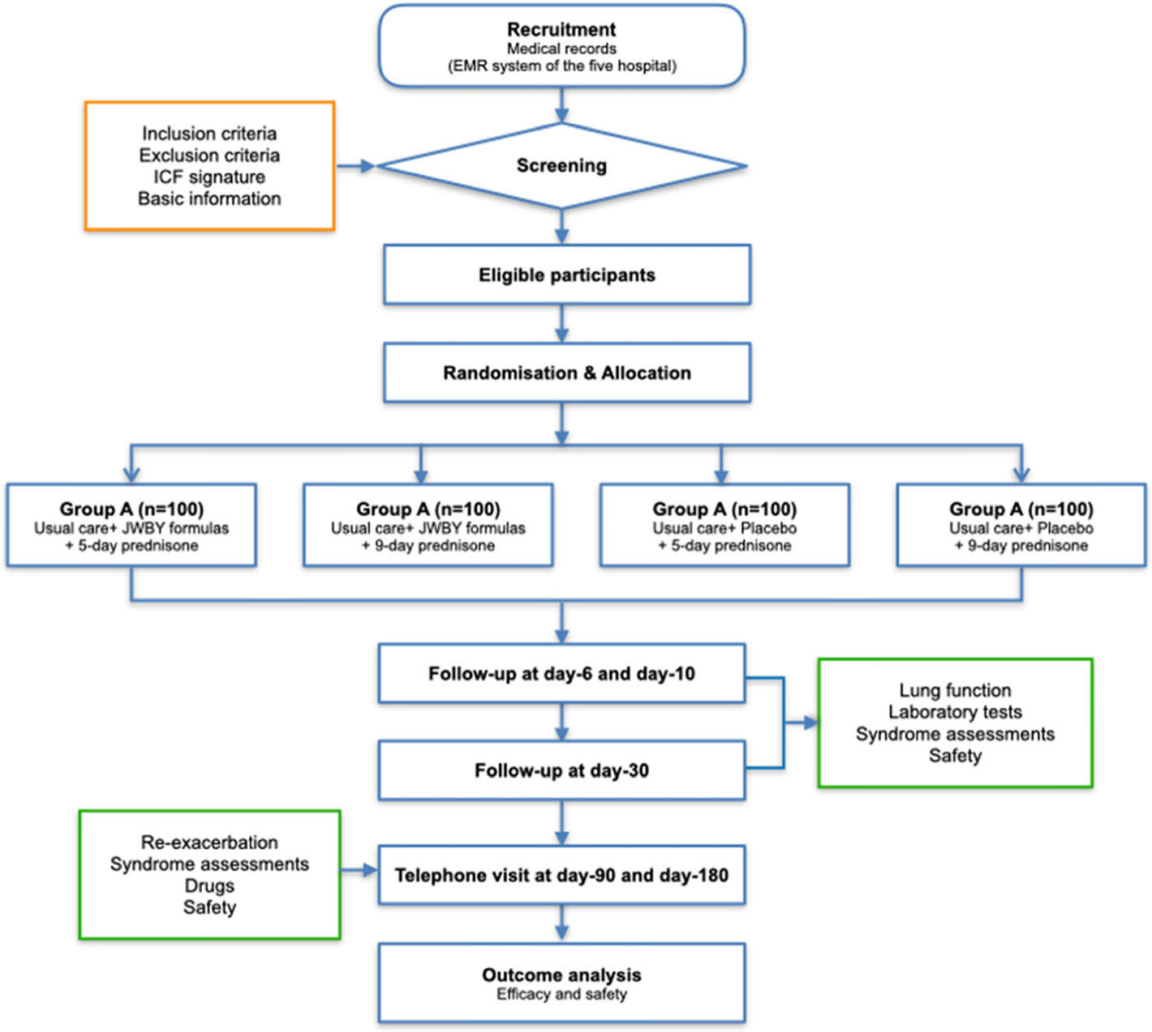
Flow chart of the study

This is a multi-center, double-blinded, placebo-controlled, randomized clinical trial. This trial will be conducted in two stages: a 9-day treatment and then a 180-day follow-up. Qualified patients will be randomized to 4 groups: group A will receive placebo plus 5-day prednisone, group B will receive placebo plus 9-day prednisone, group C will receive JWBY formulas plus 5-day prednisone, and group D will receive JWBY formulas plus 9-day prednisone. Assessments will be performed on day 6 and on day 10 during treatment and telephone calls will be conducted on day 90 and on day 180 when patients are discharged (Fig. 1).

The 9 day-treatment is chosen for two reasons. First, it is because of the two aims that were mentioned above. Second, the 9-day treatment period is based on our investigation result that most COPD exacerbation symptoms can be alleviated within 10 days. In other words, 9 days are the common hospitalization time in ten sub-centers. Therefore, the 9 day-treatment is a good time for patients to complete the study during hospitalization, which will promote the compliance of patients and collect as much data as possible.

The 180-day follow-up time is based on the results from the REDUCE randomized clinical trial research published on JAMA in 2014. It is reported in this trial that the median number of days of follow-up was 180 in both the conventional group (10th percentile, 179; 90th percentile, 181 days) and in the short- term treatment group (10th percentile, 178; 90th percentile, 181 days).

### Study setting {9}

This trial will be conducted at ten hospitals located in Shanghai, Yunnan, Xinjiang, and Jiangsu province in China. Five hospitals are selected because they are attached to universities and another five hospitals are selected because they are experienced in RCT. Also, these hospitals are spread out throughout China (Table 1). The principal investigator (PI) work at Huashan hospital and is responsible for the Steering Committee Meeting, which includes protocol training, supervision of safety, quality control, feedback of progress, and study reports. PIs of other hospitals will organize their clinical physicians and nurses to carry out recruitment and follow-up.

**Table 1:** The hospitals participating in this study

Code	Participating hospitals	Location
01	Huashan Hospital Affiliated to Fudan University	Eastern China
02	The First Affiliated Hospital of Nanjing Medical University	Eastern China
03	Affiliated Hospital of Traditional Chinese Medicine of Xinjiang Medical University	Northwestern China
04	Xinjiang Production and Construction Corps General Hospital	Northwestern China
05	The First People’s Hospital in Kashgar	Western China
06	The Second People’s Hospital in Kashgar	Western China
07	Puer Hospital of Traditional Chinese Medicine	Southwestern China
08	Yancheng Hospital of Traditional Chinese Medicine	Eastern China
09	Affiliated Hospital of Nanjing University of Chinese Medicine	Eastern China
10	Qingpu Traditional Chinese Medicine Hospital Attached to Institute of Integrative Medicine, Fudan University	Eastern China

### Eligibility criteria {10}

Patients that are hospitalized with COPD acute exacerbation and meet the inclusion and exclusion criteria (Table 2) will be eligible to be study participants.

**Table 2:** Inclusion and exclusion criteria

**Inclusion criteria**	
1. Age is between 40–90 years old	
2. Weight is between 45–85 kg	
3. Acute exacerbation of COPD with clinical grade 1–2 that only requires ordinary hospitalization	
4. Syndrome differentiation as Fei_Shen_Qi_Yu_Re Zheng in TCM	
5. Voluntarily signed informed consent	
**Exclusion criteria**	
1. Patients who also have bronchial asthma, bronchiectasis, active tuberculosis, pulmonary fibrosis, pneumothorax, pleural effusion, pulmonary embolism, or neuromuscular disease affecting respiratory function	
2. Patients who also have leukemia, aplastic anemia, myelodysplastic syndrome, thrombocytopenia, multiple myeloma, or other blood diseases	
3. Patients who also have malignant tumors	
3. Patients who also have rheumatic immune diseases and adrenal insufficiency or patients requiring hormones or immunosuppressants	
4. Pregnant or planning to become pregnant or lactating women	
5. Severe impairment of heart, liver and kidney function (heart function 3–4 degree, aspartate aminotransferase (ALT) and/or alanine aminotransferase (AST) exceeds 1.5 times of the upper limit of normal, creatinine (Cr) exceeds the upper limit of normal)	
6. Received systemic glucocorticoids within 2 weeks or participation in other drug clinical trials within 3 months prior to the trial	
7. Other conditions that the investigators consider to be improper	

Acute exacerbation of COPD with clinical grade 2 is defined as follows: respiratory rate > 30 times/min, application of assisted respiratory muscles, no mental state change, hypoxemia can be improved by the 25%–30% oxygen concentration in the inner cover of the Venturi, and hypercapnia or partial pressure of carbon dioxide (PaCO_2_) increases to 50–60 mmHg from the baseline value. Patients who are diagnosed as having respiratory failure but without the risk of death are appropriate for ordinary hospitalization, as recommended by the Chinese Expert Consensus on the Diagnosis and Treatment of Acute Exacerbation of Chronic Obstructive Pulmonary Disease (AECOPD) (2017 Update) [10]. In other words, a moderate degree of COPD exacerbation does not indicate the need for intensive care unit (ICU) admission according to 2019 GOLD Guideline [24].

TCM syndrome differentiation—Fei_Shen_Qi_Xu_Yu_Re Zheng in Chinese—specifies people who have lung and kidney qi deficiency mixed with blood stasis and heat syndrome. The diagnosis standard utilizes the 2011 COPD TCM Diagnosis and Treatment Guide and Chinese Medicine Clinical research of new drugs, in combination with previous clinical practice to develop a primary and secondary syndrome differentiation for this trial. If patients have two of the primary symptoms and two of the secondary symptoms, the TCM syndrome is determined (Table 3).

**Table 3:** TCM syndrome difference

**Primary symptoms**	
(1) Wheezing, shortness of breath, and deterioration with movement	
(2) Fatigue, or spontaneous sweating, and aggravation when in movement	
(3) Easy to catch cold, fear of wind	
(4) Quantity of sputum is yellow or sticky	
(5) The face or lips look purple and blue	
**Secondary symptoms**	
(1) Fever or thirst, like cold drink	
(2) The waist and knees are sore and weak	
(3) Tinnitus, dizziness	
(4) Incontinence or heavy urine volume	
(5) Pale or spotted tongue with yellow fur, slipping quick pulse	

Study centers are selected from level A hospital in China. The investigators will be selected from attending physician who majors in respiratory disease. Prior to the trial, all sub-center physicians, nurses, and other staff will be trained to understand the protocol.

### Who will take informed consent? {26a}

Attending physician who will take charge of the patients obtains consent from potential participants or authorized surrogates. Firstly, attending physician will introduce the trial including the origin of TCM formula, the prednisone effect, what they should do, and what will benefit them if they volunteer to participate in this trial. Then, physician will reply to the questions that confuse patients. Finally, both the physician and patient will sign the informed consent form to indicate the patient’s full understanding of the protocol.

### Additional consent provisions for collection and use of participant data and biological specimens {26b}

In the consent form, participants will be asked if they agree to use of their data should they choose to withdraw from the trial, and if they are volunteer to provide another 25 ml blood for storage, which are used to explore their inflammation level, HPA axis function, and the relationship between effectiveness and gene type. Participants will also be asked for permission for the research team to share relevant data with people from the hospitals who take part in the research.

## Interventions

### Explanation for the choice of comparators {6b}

As we mentioned in background, prednisone of 30–40 mg once daily is recommend for COPD exacerbation management since 2014 by GOLG guideline. The evidence is from a clinical trial that compare the efficacy of 14 days of prednisone treatment with 5 days. The participants come from Sweden. As in other countries like China, the duration of prednisone treatment is recommended as 9–14 days. The differences of the outcomes between 5 days of treatment and 9 days are unknown in the Chinese patients. Since we choose the relatively mild patients with COPD exacerbation, the minimum dose of prednisone 30 mg once daily is decided in this trial. In addition, TCM formula has been used for COPD therapy for thousands of years. We have observed the superiority of TCM as an adjunct therapy in COPD administration. But there is no evidence to show the exact outcomes. The doses of five TCM herbs are decided by a group of experienced TCM physician who used the principle of TCM in treating COPD for many years. The control group is placebo that contains 10% true herbs with the same appearance and smelling as the drugs.

### Intervention description {11a}

All the participants will be provided with standard of care (SOC) according to the 2019 GOLD guideline for COPD exacerbation during hospitalization and after discharge (Table 4). A 9-day adjunct medication includes systemic glucocorticoids and TCM herbs or their placebo. A basic dose of 30 mg prednisone daily for 5 days will be provided for all participants. The prednisone will be continued in the long-term glucocorticoids arm of the trial in the following 4 days and replaced with the placebo in short-term glucocorticoids arm of the trial. The 9-day treatment period is based on the fact that most COPD exacerbation can be relieved within 10 days.

**Table 4:** SOC and treatments

**During hospitalization**	
Oxygen therapy	Continuous low-flow oxygen absorption (oxygen concentration is 2 L/min).
Short-acting inhaled bronchodilator	Albuterol atomization solution 5 mg, ipratropium bromide atomization solution 500μg, nebulization, Bid.
Theophylline	Aminophylline 0.5 g for injection, intravenous drip, Qd; Peony ambroxol 60 mg, intravenous drip, Bid.
Antibiotics	Penicillins: piperacillin tazobactam 4.5 g, intravenous infusion, Q8h; quinolines: levofloxacin injection 0.5 g, intravenous infusion, Qd. Antibiotics for 7–10 days.
**After discharge**	
Group A use a bronchodilator	
Group B use LAMA or LABA	
Group C use LAMA	
Group D use LAMA and/or LABA+ICS	

Group A to D is classified according to the 2019 GOLD guideline

*LAMA* long-acting anti-muscarinic antagonists, *LABA* long-acting beta2-agonists, *ICS* inhaled corticosteroids

Meanwhile, participants will be treated with TCM herbs or placebo. Participants will be randomized to four groups with different adjunct medication (Fig. 1). Because of the complex and variety in COPD exacerbation, variation among patients will be allowed. Any variation like another antibiotic used for the indication will be recorded in the case report form (CRF).

#### TCM treatment

TCM treatment is in accordance with the most common TCM syndromes of COPD in a real-world study [40]. The dosage of JWBY formula is selected according to the pharmacopeia of Chinese medicine, and the effective ingredient of its granules is determined according to the pharmacopeia of pharmacopeia.

JWBY formulas contain 5 kinds of herbs: Huang Qi (Astragalus) 30 g, Yin Yang Huo (Epimedy) 20 g, Sheng Di Huang (Radix Rehmaniae) 15 g, Chi Shao (Red Peony) 30 g, and Huang Qin (Scutellaria) 30 g, concentrated as 20.48 g granules. To use, patients can infuse 10.24 g granules into 125 mL of boiling water and ingest orally after breakfast and supper, twice daily. Its placebo is identical in appearance, shape, size, and package with JWBY formulas, but only contains 10% real herbs. The granules will be produced and packed by Huarui Sanjiu pharmaceutical industry in Shenzhen, China. Granule production will be certified to get the standard certification of the TCM National Drug Regulatory Authority.

### Criteria for discontinuing or modifying allocated interventions {11b}

Modification or discontinuation of the intervention will be decided by the PIs in each center, according to the requests from participants, or when a participant’s disease is worsened to grade 3 which indicates the need for ICU admission, or when unexpected adverse effects happen.

### Strategies to improve compliance to interventions {11c}

Prednisone and JWBY granules are free as study drugs. Five-day drugs will be provided to participants at baseline by a sub-center investigator and another 4-day drug at day 6. Participants will use patient diaries for recording medication and changes in symptoms. All unused packs of drugs and empty bags will be returned to investigational site on day 6 and on day 10. Compliance will be calculated by counting drugs or empty bags for a 9-day course. Compliance % of medication = [actual dose/(specified daily dose × days)] × 100%. Total medication consistency ranging from 80 to 120% will be eligible for the protocol analysis set. Patients enrolled in the trial will be all hospitalized and all the laboratory tests will be performed on standard schedule, which aids in the monitoring of adherence. Once the patient is randomized, the investigators will take every reasonable effort to follow the patient for the entire course of the study. All examination and transportation costs in the 30-day will be covered and the results of symptoms and physical exams will be explained at every visit. Messages will be sent through WeChat or by phone prior to every visit to remind the patients of the follow-up visits.

### Relevant concomitant care permitted or prohibited during the trial {11d}

Extra COPD-related drugs, such as leukotriene receptor antagonists, antihistamines, immunosuppressants, and antioxidants, will be forbidden during the trial. TCM herbs that are tonifying kidney, benefiting Qi, clearing away heat, and promoting blood circulation, whose TCM characteristics are like those within JWBY formulas, will be avoided. Drug combinations will be recorded in the case report form at each follow-up visit.

“Tonifying Kidney” (“Bushen” in Chinese) is a TCM term of treatment, which aims at the TCM syndrome “deficiency of Kidney” (“Shen_xu” Zheng in Chinese). The Chinese herbs used in “Tonifying Kidney” treatment can relieve “deficiency of Kidney” syndrome including shortness of breath, deterioration with movement, fatigue, waist and knee area sore, and their weakness, tinnitus, dizziness, incontinence, or heavy urine volume.

### Provisions for post-trial care {30}

Patients that are enrolled into the study will be covered by indemnity through the standard National Health Service Indemnity arrangements. The PI will provide the compensation to those who suffer due to trial participation.

### Outcomes {12}

#### Primary outcomes measurements

1. The time to the next exacerbation of COPD during the 180-day follow-up is defined as one primary outcome. The definition of exacerbation is deterioration of the cardinal symptom of dyspnea, increased sputum purulence and volume, and purulent sputum. This may be combined with one of the other symptoms: increased cough and wheeze, sore throat, nasal congestion due to cold, fever (oral temperature > 37.5 °C), increased cough, and increased wheezing. The above changes should last for ≥ 2 days at least. A minimum of 1 week between two exacerbations is needed in order for them to be considered as separate events. The duration of exacerbation is measured from the onset of acute exacerbation to a significant reduction which is defined as the symptoms return to the level before the exacerbation per the records in patients’ dairies. The diaries are distributed to participants during the treatment and after the treatment. Participants record changes of their symptoms and their health status by choosing the right description in terms of feeling. The primary symptom is measured with modified British Medical Research Council (mMRC) and COPD assessment test (CAT) scores. The days of exacerbation are calculated from the onset date of the primary symptom to the date when all symptoms disappear. The degree is classified as mild (treated with short acting bronchodilators only, SABDs), moderate (treated with SABDs plus antibiotics and/or oral corticosteroids), or severe (patient requires hospitalization or visits to the emergency room). Severe exacerbations may be associated with acute respiratory failure.
2. The mean difference of CAT scores between day 6 or day 10 and baseline is another primary outcome. The CAT involves an 8-dimension measurement of health-status impairment in COPD. CAT is universally acknowledged as a reliable and valid measurement in evaluating the changes of COPD.

#### Secondary outcomes measurements

1. TCM syndrome assessment will be evaluated from baseline to day 6 and day 10. According to the Guiding Principles for Clinical Research of New Drugs in Traditional Chinese Medicine, the syndrome score is calculated as efficacy index *n* = (pre-treatment score − post-treatment score)/pre-treatment score ×  100%. In terms of mild, moderate, and severe symptoms, the primary symptoms are given 2, 4, and 6 points while the secondary symptoms are given 1, 2, and 3 points respectively. Total score = scores of the primary symptoms + scores of the secondary symptoms.
2. Lung ventilation function will be assessed by forced expiratory volume in 1 s (FEV1), forced vital capacity (FVC), and peak expiratory flow (PEF) from baseline to day 6 and day 10 with standardized equipment (Erich Jaeger UK Ltd., Market Harborough, UK Jaeger Master-Screen, Germany) and per the standard procedure recommended by American Thoracic Society (ATS) [39].
3. Blood gas analyses including partial pressure of oxygen (PaO_2_), partial pressure of carbon dioxide (PaCO_2_), infectious indexes including blood eosinophil count in cells per micrometer (EOS), C-reactive protein (CRP), and proclamation will be tested by clinical laboratories in the sub-center from baseline to day 6 and day 10.

#### Safety outcomes

Side effects will be collected at day 30, day 60, and day 180 during follow-up. This specifically refers to (1) the changes in hyperglycemia: fasting plasma glucose ≥ 5.6 mmol/L or random plasma glucose ≥ 7.8 mmol/L or rise ≥ 20% in daily doses of insulin or any increase in oral anti-diabetic drugs or initiation of one or more anti-diabetic therapeutics, (2) changes in hypertension: systolic blood pressure ≥ 140 mmHg and/or diastolic blood pressure ≥ 90 mmHg or the addition of one or more anti-hypertensive drugs to previous treatment regimens, and (3) the number of psychiatric symptoms, asphalt, vomiting coffee samples, and new infection.

Laboratory tests which include routine blood test, routine urine test, electrocardiogram (ECG), kidney and liver function, and X-ray computed tomography (CT scan/X-ray) of the chest will be conducted at baseline, day 10, and day 30 during the follow-up. If the results of CT scan/X-ray and ECG are normal at baseline, it will be skipped in the follow-up.

#### Exploratory outcomes

The pathology of COPD is relevant to the inflammation and the suppression of the HPA axis that follows the treatment with glucocorticoids. Therefore, changes in the HPA axis including corticotropin-releasing hormone (CRH), adrenocorticotropic hormone (ACTH), and cortisol and the inflammation cytokines including interleukin-6, interleukin-8, and interleukin-10 at baseline and on day 6 and day 10 will be measured.

### Participant timeline {13}

#### Sample size {14}

There are four groups with two variables in this trial—TCM treatment and systemic glucocorticoid treatment. Therefore, according to primary endpoints collected from previous trial [34, 41], we choose the maximum sample size needed, as calculated by two way on http://www.powerandsamplesize.com (Table 2). At the 5% significance level, a total of 67 patients per group will be required for a 2-group, 1-sided calculation to achieve 80% power and the differences of 10.30 ± 6.31 and 12.95 ± 5.99 in CAT mean score between the TCM treatment group and placebo group (Table 5). Meanwhile, a total of 88 participants will be required for a 2-group non-inferiority calculation to achieve the mean difference of the time to next exacerbation (43.5, 29) and a non-inferiority margin of 10, under the condition that the standard deviation of the groups is equal to 12 (Table 5). A loss of 15–20% to follow-up is predicted based on experience—this increases the sample size to 200 participants per group, resulting in 400 in total.

**Table 5:** Estimation of sample size

Variable	TCM treatment	Systemic glucocorticoid treatment
Primary outcome	CAT score	Time to next exacerbation
Way	2-group,1-sided	2-group non-inferiority
Group A mean	10.30	29
Group B mean	12.95	43.5
Group A standard deviation	6.31	12
Group B standard deviation	5.99	12
Non-inferiority margin		10
Sampling ratio	1	1
Type 1 error	0.05	0.05
Power	0.8	0.8
Sample size	67	88

### Recruitment {15}

All investigators in the sub-center will advertise and distribute posters in their emergency department and nearby communities. In addition, we will set up a hierarchical medical system in Shanghai—communities refer the potential patients to Huashan hospital directly where the clinical trial is undertaken.

## Assignment of interventions: allocation

### Sequence generation {16a}

Participants will be randomized with equal probability (1:1:1:1) to receive one of the four treatments that were mentioned above. As the size of each group is predicted to be 100, the allocation sequence is generated with sample randomization and stratification by trial center. The sequences will be generated by software and in Excel format.

### Concealment mechanism {16b}

Before the study begins, a series of random numbers will be generated by the computer, and the pharmacists involved in the study place the random numbers in plain, closed envelopes marked with patient numbers. Envelopes will be made and stored at the pharmacy and opened by the pharmacist only when the subjects are randomized. The envelopes will be not accessible to individuals directly involved in the study.

### Implementation {16c}

Allocation sequence will be generated by a statistician who will not participate in enrolling participants. Participants will be blindly randomized and allocated with an identified number. Principal investigators including attending physician and nurses will involve in enrolling participants. Pharmacist will distribute an independent emergency envelope for each participant, which contains the treatment assignment. The participants in the placebo group will be given the same number of pills and followed the same medication schedule as the treatment group. To ensure the implementation of the blinding method, the pill and herbs in both the treatment group and the placebo group will be made in the same shapes, smells and tastes.

## Assignment of interventions: blinding

### Who will be blinded {17a}

Trial participants, care providers including attending physician and nurse, outcome assessors including PI and sub-PI, and data analysts will be blinded after the assignment of interventions. Double-grade unblinding will be adopted. First grade unblinding: it will be conducted before the data analysis. After the double input of all the CRF data into the computer and blinded review, the data will be locked. Afterwards, the personnel who keep the blinded materials will unblind them for the first time, which is to divide the groups corresponding to the case numbers into blinded codes of two groups and to tell the statisticians so as to statistically analyze all the data. Second grade unblinding is after the statistical analysis and the completion of clinical trial report. It will be conducted at the wrap up meeting for the clinical trial. The treatment group and control group will be unblinded. Place of unblinding will be the unit where the clinical trial is in charged. Executive personnel will be the chief researcher and statisticians of the unit that are in charge of the trial.

### Procedure for unblinding if needed {17b}

If there is severe adverse event, which impedes the progress of the trial and the selection of the treatment measures, urgent unblinding can be carried out. During the process, all the researcher, sub-PI, and clinical supervisors should take part. The local administrative unit should be informed within 24 h. The reason, time, and place of unblinding should be recorded in detail and all the records should be signed off. Afterwards, the clinical supervisors should be informed timely. The case data should be kept intact.

## Data collection and management

### Plans for assessment and collection of outcomes {18a}

Prior to the start of the trial, sub-center physicians will be trained. The results of laboratory tests from different hospitals are adjusted per the Huashan Hospital standards during analysis. Demographic information (date of birth, gender, etc.) and medical condition (medical history, concomitant medication, etc.) will be recorded at baseline. All the questionnaires will be answered by patients without inducement. When adverse events that are related to study drugs happen, emergency envelope can be considered as needed to be opened by PIs and physician. The investigators will report the reasons and outcome to the PI within 24 h.

### Plans to promote participant retention and to complete follow-up {18b}

**Fig. 2:**
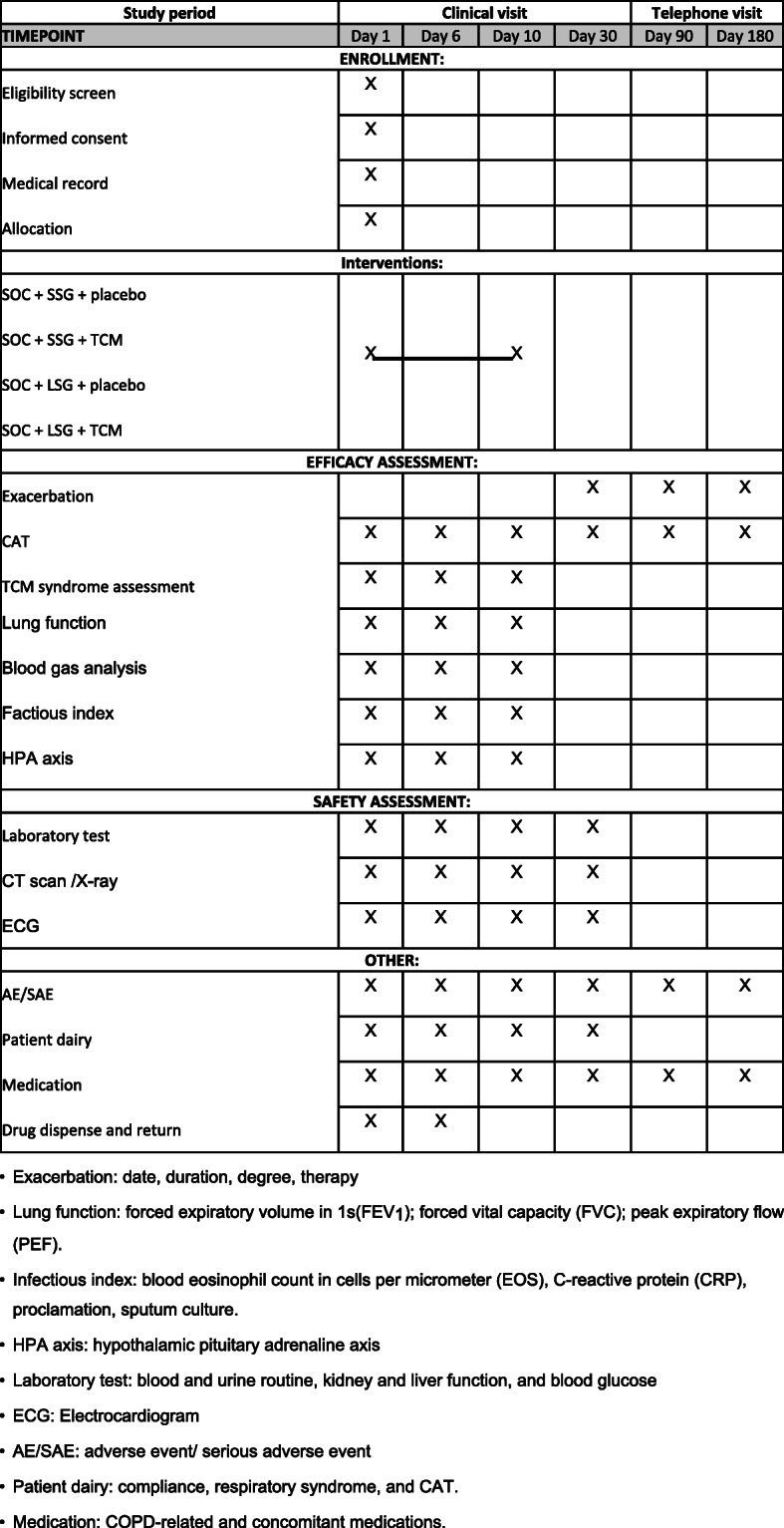
Schedule of enrollment, interventions, and assessments

Prior investigation shows that the mean hospitalization duration time is about 8–9 days in these 10 hospitals, which matches the trial requirement of 9 days of treatment. After screening and completing baseline evaluations, participants will visit the physician at day 6 and day 10 during adjunctive treatments and day 30 when patients are discharged (Fig. 2). We will provide free TCM granules and partial examination reimbursement to participants. The participants and their family member will be informed that standardized treatment is beneficial to reduce COPD exacerbation, which will reduce medical expenses the benefits. Two telephone calls will be conducted on day 90 and day 180.

### Data management {19}

#### The writing and transfer of case report

The case report will be written by the doctor who has participated in the trial. Every case should have a complete case report. The case report, once completed, should be checked by the supervisor. Afterwards, it will be transferred to the data administrator for data entry and management.

#### The design and establishment of database

All the information in CRF table will be recorded in a specialized clinical experimental database that is designed by Chinese academy of Traditional Chinese medicine. The format of the database should be close to that of the CRF table so as to facilitate the data entry.

#### Data coding

The variables in the CRF table will be encoded and the codes will be kept unchanged during the whole process of clinical research.

#### Data entry

The CRF data will be entered by highly trained specialists from the research centers.

#### The audit of data

The audit of data can be divided into two forms: manual audit and system audit. The former refers that the administrator checks the consistency and logic of the data so as to find the mistakes and to generate the question list. SAS software sets the limit of all variables and rules out automatically the unqualified data by running the system program. The question list is sent to the clinical supervisor who transfers it to the researcher for reconfirmation. The related revision should be signed and dated by the researcher.

#### Data locking

The researcher will correct the data for the last time after the return of all question lists. All the corrections and updates should be recorded and filed. After the data is verified, the data administration meeting will be held so that the corrections and updates can be summarized. At last, the data administrator will announce the locking of database and keep the cipher code. The statistical analysis prospectus will not be changed after the database lock. The data will be transferred to the statistical department for analysis.

#### Data storage

All the data should be kept according to the requirements of GCP. After the experiment, all the original copy of case reports and records for the administrations of clinical drugs should be checked, signed, and stamped by the supervisors, head researchers, and representatives from GCP office of each clinical center, and finally, these records will be sent to the leading site where the database will be established and the data will processed. Statisticians will analyze the data and materials from the participating centers, and the summary of the clinical trial will be completed in the leading site.

### Confidentiality {27}

Case report form (CRF) collects all the information throughout the trial for every participant. As soon as verification is completed, data will be securely stored and sent to Huashan Hospital from the sub-centers. A data management group will be established, and the information will be entered into the database provided by http://www.rilintech.comt through independent double-data entry. The errors and inconsistencies of data will be checked during the entry process. The user identification code and password will be protected by the data management group. The PIs will be given access to the cleaned data sets. Sub-investigators will only have access to the data sets in their own hospital. Original paper forms will be kept in Huashan Hospital for 5 years.

### Plans for collection, laboratory evaluation, and storage of biological specimens for genetic or molecular analysis in this trial/future use {33}

#### The process and collection of blood samples

##### Separation of 1–2 ml of plasma from 4 ml of whole blood

The anticoagulant and aprotinin (for concentration and amount, please refer to the note) will be added to the blood-collection tube, which is placed at 4 °C for precooling, and then1.5 ml of whole blood will be collected. The samples will be mixed in the tube slowly, and afterwards, the mixture will be centrifuged at a low temperature (4 °C, 4000 r/min, 15–20 min). 0.5 ml of plasma will be collected and kept at a low temperature (− 80 °C). If the collected blood cannot be centrifuged immediately, it can only be stored in 4 °C freezer for up to 1 h.

Note: The concentration and amount of anticoagulant and aprotinin.

Anticoagulant: 0.3MEDTA.2Na concentration (20ul/ml) or 1% heparin (10ul/ml);

Aprotinin (500 IU/ml). There are two kinds of aprotinin: liquid (the concentration will be noted on the label) and solid (10,800 IU/mg). The solid form of aprotinin can be dissolved in normal saline, so its concentration can be adjusted to 500 IU/20 μl.

##### Requirements for sample storage

The samples should be kept immediately in − 80° freezer. Throughout the transportation, the samples cannot be taken out. In Huashan Hospital, all of the samples are checked. The samples should be labeled with case codes and collection date. Blood serum should be kept in dry ice for transportation.

### Statistical methods

#### Statistical methods for primary and secondary outcomes {20a}

##### Primary and secondary outcomes

The TCM intervention arm—JWBY (Jia Wei Bushen Yiqi formulas)—will be compared against the placebo. The short-term systemic glucocorticoid (SSG) arm will be compared against the long-term systemic glucocorticoid (LSG) arm. Four groups will be compared with each other independently.

Statistical Package for Social Sciences for Windows, version 24.0 (SPSS, Chicago, IL, USA) will be used for analysis. The tests will be 2-sided, and a *P* value with alpha ≤ 0.05 level is considered significant. *P* values will be reported to four decimal places with *P* values less than 0.001 reported as *P* < 0.001. The Bonferroni method will be used to appropriately adjust the overall level of significance for multiple comparisons, assuming an exchangeable correlation structure. Categorical variables will be summarized by absolute numbers and percentages of total. The difference of categorical variables will be assessed with the generalized estimating equations (GEE). GEE will also be used to assess the impact of potential clustering of participants in the same hospital. Safety outcomes will be analyzed with summary statistics (frequency, count, percentage).

The method of analysis of each variable are summarized in Table 6.

**Table 6:** Variables, measures, and methods of analysis

Outcomes	Hypothesis	Outcome measurement	Analysis method
**Primary**	**TCM improved outcomes from baseline to 10 days**		
Interval time to next exacerbation		Telephone visit (continuous)	GLM
CAT		Questionnaire score (continuous)	MENM
**Secondary**	**Improved occurred**		
Duration of COPD exacerbation		Calculating days in dairy (continuous)	GLM
TCM syndrome assessment		Questionnaire score (continuous)	GLM
Lung function		FEV1, FVC, PEF (continuous)	GLM
Blood gas analysis		Pa (CO_2_), Pa(O_2_) (continuous)	GLM
Infection index		EOS count, CRP, proclamation (continuous)	GLM
Inflammation cytokine		IL-6, IL-8, IL-10(continuous)	GLM
HPA axis		ACTH, CRH, CORT (continuous)	GLM
**Subgroup analysis**	**Effects adherence**	**Groups**	
Age (in years)		< 64, ≥ 64	
Gender		Male, female	
Body mass index (kg/m^2^)		< 18.5, ≥ 18.5 to < 24, ≥ 24	
Smoking history		Ex-smoker, current smoker	
GOLD stage		I-II, III, IV	
Infectious		Infectious, un-infectious	
EOS count		< 300/μl, ≥ 300/μl	

The score of COPD assessment test (CAT) will be collected at baseline, day 6, day 10, and in the 30 days, 90 days, 180 days after discharge. The mixed effect normal model (MENM) will be used to compare each outcome against the TCM intervention group and placebo. The estimate of treatment effect will be presented as unadjusted rate ratio followed by an adjusted ratio with adjustment for a set of pre-specified baseline variables. The list of pre-specified variables is as follows: centers (as a random effect), age (in years), gender (male or female), weight (in kilogram), smoking (pack per year), FEV1% predicted, the number of COPD exacerbation in the previous 1 year, and home-oxygen therapy. Fixed effects will include the visit number, treatment, and all the pre-specified variables. Participant and visit interaction will be fitted as random effects. An autoregressive correction structure will be used throughout. The difference of interval time to next exacerbation during follow-up in the 30 days, 90 days, and 180 days will be compared between SSG and LSG groups using the generalized linear model (GLM) with a log-link function, a propriety over dispersion parameter, and length of time as an offset. The numbers will be described respectively in three grades—outpatient, inpatient, and ICU.

Durations of COPD exacerbation will be compared between each two of the four groups with GLM in the similar manner as before. Specially, the shortness of breath measured by mMRC dyspnea scale (1–5 degree) in the diary will be undertaken in the Logit link function independently.

The changes in TCM syndrome score, infectious index, lung function, blood gas analysis, inflammatory cytokine levels, and HPA axis will be collected in baseline, at day 6, and at day 10. GLM will be used to analyze the change between each two of the four groups as well.

#### Interim analyses {21b}

None. In this trial, interventions for participants include 9 days of TCM granules and 5 or 9 days of prednisone. These two interventions will be carried out during hospitalization and they are routine treatments in China, so there are no anticipated problems that will be detrimental to the participant. Therefore, there will be no interim analyses and there are not anticipated formal stopping rules for the trial.

#### Methods for additional analyses (e.g., subgroup analyses) {20b}

##### Subgroup analysis

The potential subgroups have been listed in Table 3. The analysis of primary outcomes will be repeated in the subgroups.

#### Methods in analysis to handle protocol non-adherence and any statistical methods to handle missing data {20c}

Analysis will be in accordance with the intent-to-treat principles. The safety set (SS) includes participants that are randomized and have received adjunct treatments and one post-treatment safety assessment at least. The full analysis set (FAS) includes participants that are randomized and have received adjunct treatment, and their primary outcomes are available at least in one visit. The per protocol set (PPS) includes participants in accordance with all the following conditions: valid baseline values, compliance with the program, no violation of the inclusion and exclusion criteria specified in the program, completion of all assessments, and good compliance (defined as participants taking at least 80% of expected doses of study drugs as determined by counting).

Missing data is predicted to appear on day 30 and during the two telephone calls after discharge. The imputation of all the outcomes will be replaced by the mean of the group.

### Plans to give access to the full protocol, participant level data, and statistical code {31c}

Full protocol participant level dataset in Chinese will be accessible in the register site. And statistical code will be provided by trial statistician. For sharing purpose, data will be available to outside investigators at the end of the trial. The finding of this trial will be published in peer-reviewed journals and presented at conferences. The results of the study will be released to the participating physician and patients.

## Oversight and monitoring

### Composition of the coordinating center and trial steering committee {5d}

Multi-center trial coordination committee will be established. The Huashan Hospital Affiliated to Fudan University will take charge of the committee, and the main researchers of the participating units will serve as the members. The committee will be responsible for the implementation of the whole experiment and resolve problems during the trial process. The head researcher should strengthen quality surveillance of the clinical trial in his own center.

### Composition of the data monitoring committee, its roles and reporting structure {21a}

The data monitoring committee is unnecessary in this trial, because the drug duration in this trial is short—9 days. TCM granules and prednisone are routine treatment in China and will be carried out during hospitalization; only minimal risks are anticipated.

### Adverse event reporting and harms {22}

#### Adverse event report

Regardless of whether it is related to the study drug or not, any clinically significant abnormalities of medical events or laboratory tests will be defined as an adverse event (AE). For all adverse events, the time, duration, treatment measures and outcomes, the severity of the disease, and the association with the study drug will be evaluated and recorded. It is divided into mild, moderate, and severe according to the following list: conscious symptoms, ability to tolerate, impact on daily activities, duration, whether it is relieved during continued medication, and whether treatment is required. Serious adverse events (SAE) will be defined as death or life-threatening events. If a SAE occurs, the doctor will immediately take emergency measures and report it to the PI and the ethics committee within 24 h.

#### Evaluating the association with drugs

According to the occurrence of adverse events and a reasonable time interval, and alleviation after withdrawal of the study drugs, the correlation between adverse events and study drugs will be evaluated as affirmative (sure), probably related (very likely), may be relevant (possible), may be unrelated (suspicious), and irrelevant (impossible). Due to the unsatisfactory treatment effect, the patient will withdraw from the trial. The emergency letter of the case will be opened, and the patient’s family will coordinate with the follow-up and report the result to the lead center. The relevant information will be recorded in the case report form.

#### Gastrointestinal reaction

Although the formula is optimized to instant granule instead of TCM herbs decoration in our study, some participants who never accepted TCM herbal previously may have gastrointestinal reactions such as nausea and vomiting. They will be suspended for 3 days and evaluated on their abilities to continue to participate in.

#### COPD deteriorating

Because the participants are in the acute exacerbation period, their disease may deteriorate to grade 3 at any time with the worsening of clinical symptoms including increase of dyspnea, mental consciousness changes, blood gas analysis of acidosis, and hypoxemia that cannot be improved by oxygen absorption or other treatments. Participants will be admitted to the intensive care unit (ICU) if it happens. Due to the worsening of the disease or the unsatisfactory effect, the emergency letter of the case will be opened. The physician-in-charge will communicate with the patient’s family if participant needs to withdraw from the study. The relevant information is reported to PI and recorded in the case report form. As for any deterioration syndromes that arise after discharged, participants will be advised to come to the hospital. The investigator will provide free medical services appropriately.

#### Glucocorticoid’s withdrawal

The recommend dose of prednisone by 2017 Chinese consensus is 30–40 mg daily for 9–14 days. The low dose of 30 mg is chosen. Extra management measures were suggested during the initial meeting. First, the participants will be informed that the withdrawal symptoms include fatigue, joint muscle soreness, low mood, poor appetite, and even nausea and vomiting. Second, participants discharged from the hospital with adrenal insufficiency will receive instructions on how to take less than 30 mg daily if they cannot tolerate the treatment. Finally, participants will be advised to take the following preventive measures against possible adverse events.

1. Patients with diabetes should monitor blood glucose closely and modulate the number of hypoglycemic agents or insulin.
2. Investigators will pay attention to whether the patient has abdominal pain, vomiting of coffee-like substances, or tar-like black stool. If this occurs, the patient should promptly come to the emergency department and be treated with acid-suppressing stomach and other drugs.
3. Investigators will observe the patient’s neuropsychiatric symptoms closely, such as euphoria, excitement, mania, and insomnia. If necessary, advise patients to seek medical help.

### Frequency and plans for auditing trial conduct {23}

The designated monitor will visit each investigational site once a month. The monitor will check that if the regulatory binder is complete and all that associated documents is stored well or not, including CRF, informed consent forms, and adverse events reports. And the monitor will help the investigational site resolve the issues happened in the trial.

### Plans for communicating important protocol amendments to relevant parties (e.g., trial participants, ethical committees) {25}

Any modification to the protocol which may impact the conduct of the study and the potential benefit of the patients will be reported to ethic committee. The amendments will be approved by the ethics committee before it is announced to each investigational site. And an investigator training about new protocol will be held through WeChat video meeting. Participants will be informed of the new protocol.

### Publication plans {31a}

Participant information will not be released outside of the study without the permission of individuals except for monitoring. Blood samples, data collection, and administrative forms will be identified with the same code and stored separately in a locked place. All data will be uploaded to the ResMan original data sharing platform (IPD sharing platform) http://www.medresman.org of the China Clinical Trial Registry, which is available to outside investigators when the trial ends. The result will be published in peer-reviewed journals and shared at conferences. The findings of the trial will be released to the participating physicians and patients.

## Discussion

With the design of TCM as an adjunct to systemic glucocorticoids to treat COPD exacerbation in this randomized trial, we will test the non-inferiority of two different treatment terms of systemic glucocorticoids in COPD exacerbation. The finding will bring new proofs to the controversial applications of glucocorticoids. In addition, we will clarify a pragmatic method to identify the efficacy of classic description based on TCM syndrome differences despite limitations like bias of measurement and the subjectivity of the questionnaire assessment which may be exacerbated by the loss of some participant during follow-up. The difference between the four groups will indicate that TCM reduces the suppression of the HPA axis and strengthens the anti-inflammation effect of glucocorticoids. TCM may strongly support and enrich the management of COPD exacerbation.

However, there are some limitations in this protocol. Firstly, we choose one of the specific TCM syndromes as the criteria. The result is hard to be extended to the whole patients with COPD exacerbation. In addition, we use the Chinese guideline to evaluate the degree of COPD exacerbation, which relies on the subjective assessment of symptoms of the enrolled participants by the physician. Hopefully, an objective method will be proposed to assess the COPD exacerbation.

## Trial status

Our trial has enrolled 10 volunteers in Shanghai from 07 August 2019 up to today. We have modified protocol according to the practice and standard protocol items [42, 43]. In the meantime, we proposed amendment to ethics commitment and Chinese clinical trial registry in July of 2019. The protocol version is KY 2019-299,03,03, July, 2019. The new protocol was reported to all sub-center PIs in a group meeting. Due to COVID-19, we expect to complete the recruitment process around October 2020 and report the results as soon as possible.

## Supplementary information

**Additional file 1.**

## Data Availability

The enrolled participants who sign the informed consent forms about clinical data and bio-sample collection are provided with free medication of JWBY formulas and prednisone for 9 days as needed. Participants’ information will not be released outside of the study without the permission of individuals except for monitoring. Blood samples, data collection, and administrative forms will be identified with the same code and stored separately in a locked place. All data will be uploaded to the ResMan original data sharing platform (IPD sharing platform) http://www.medresman.org of the China Clinical Trial Registry, which is available to outside investigators when the trial ends. The result will be published in peer-reviewed journals and shared at conferences. The findings of the trial will be released to the participating physicians and patients.

## Abbreviations

COPD: Chronic obstructive pulmonary disease
AECOPD: Acute exacerbation of chronic obstructive pulmonary disease
TCMWSG: Traditional Chinese medicine with systemic glucocorticoids
TCM: Traditional Chinese medicine
JWBY: Jia Wei Bushen Yiqi
LSG: Long-term systemic glucocorticoids
SSG: Short-term glucocorticoids
SABDs: Short acting bronchodilators
mMRC: Modified British Medical Research Council
CAT: COPD assessment test
PaO_2_: Partial pressure of oxygen
PaCO_2_: Partial pressure of carbon dioxide
EOS: Blood eosinophil count in cells per micrometer
CRP: C-reactive protein
FEV1: Lung function: forced expiratory volume in 1 s
FVC: Forced vital capacity
PEF: Peak expiratory flow
ATS: American Thoracic Society
HPA axis: Hypothalamic pituitary adrenaline axis
ECG: Electrocardiogram
AE/SAE: Adverse event/serious adverse even
LAMA: Long-acting anti-muscarinic antagonists
LBMA: Long-acting Beta2-agonists
ICS: Inhaled corticosteroids
